# Next-generation non-animal models for inflammatory bowel disease: *In vitro* and *in silico* approaches for mechanistic understanding

**DOI:** 10.1016/j.namjnl.2025.100051

**Published:** 2025-09-12

**Authors:** Priyanka Raju Chougule, Sukesh Narayan Sinha

**Affiliations:** a Food Safety Division, Indian Council of Medical Research–National Institute of Nutrition, Jamai-Osmania, Hyderabad 500007, India; b Department of Biochemistry, Osmania University, Hyderabad, Telangana 500027, India

**Keywords:** Inflammatory bowel diseases, Cell culture, Organoids, Computational biology, Signal transduction

## Abstract

Inflammatory bowel disease (IBD), encompassing ulcerative colitis and Crohn's disease, is a chronic and relapsing inflammatory condition of the gastrointestinal tract resulting from a complex interplay of oxidative stress, immune dysregulation, microbial imbalance, and epithelial dysfunction. While animal models have primarily contributed to our understanding of IBD pathogenesis, their limited translational relevance and ethical concerns have led to the accelerated adoption of New Approach Methodologies (NAMs). This review highlights the increasing importance of *in vitro* platforms, including intestinal epithelial cell lines, 3D organoids, and microfluidic gut-on-chip models, which offer physiologically relevant and ethically sustainable solutions. *In silico* strategies, such as molecular docking, network pharmacology, toxicogenomics, artificial intelligence, and machine learning, enhance these models by enabling predictive simulations of drug interactions and molecular targets. Emerging mechanisms, such as barrier modulation and multi-targeted inflammatory regulation, are being explored using NAMs. Despite significant progress, challenges remain in standardization, integrating the immune and microbiome systems, and achieving regulatory acceptance. The combination of NAMs, multi-omics, and real-world data represents a promising avenue for mechanistic research, therapeutic screening, and precision medicine in IBD.

## Highlights

• NAMs offer ethical, human-relevant alternatives to animal models in IBD research.
• 2D cell models effectively replicate barrier dysfunction and inflammation.
• 3D cell models and co-cultures mimic complex intestinal microenvironments.
• *In silico* tools support drug screening, toxicity prediction, and target identification.
• Integration of NAMs with omics enhances precision medicine in IBD.

## Graphical abstract

**Figure:**
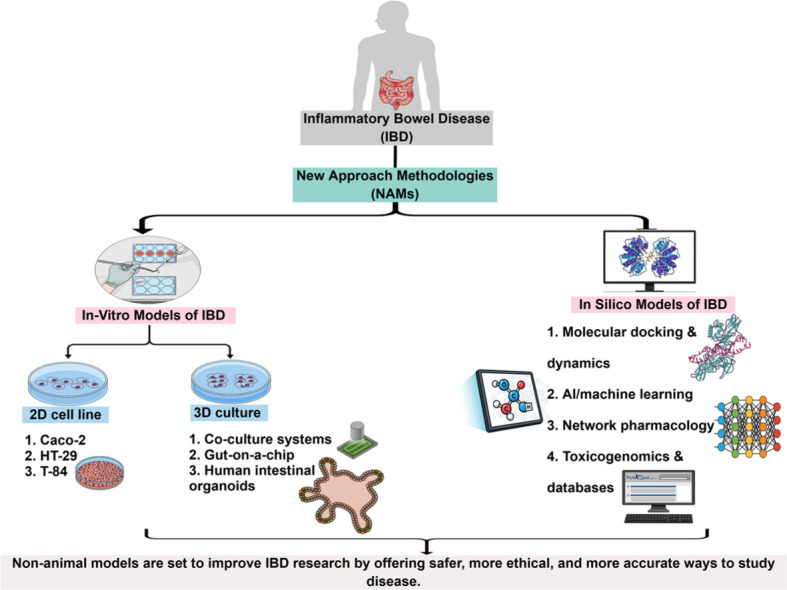

## 1 Introduction

### 1.1 Introduction to inflammatory bowel disease (IBD)

Inflammatory bowel disease (IBD) is a chronic, relapsing, and idiopathic inflammatory condition of the gastrointestinal tract, marked by elevated levels of inflammatory mediators, leukocyte infiltration, oxidative stress, disruption of the epithelial barrier, and an imbalance in the gut microbiota. IBD encompasses two major clinical subtypes, ulcerative colitis (UC) and Crohn’s disease (CD). While CD can affect any part of the gastrointestinal tract in a discontinuous pattern, often involving the ileum and colon, UC is typically confined to the rectum and colon, extending proximally in a continuous manner (pancolitis). Precise diagnosis is essential, as symptoms can overlap with other conditions such as eating disorders, including anorexia nervosa and bulimia nervosa (McClain et al., 1993).

### 1.2 Epidemiology and public health burden

The global burden of IBD has evolved considerably over the years. As of 2019, approximately 4.9 million individuals were affected worldwide, with China and the United States reporting prevalence rates of 66.9 and 245.3 cases per 100,000 population, respectively (Wang et al., 2023). In India, an estimated 31,774 new cases of IBD were reported in 2019, accounting for nearly 8% of the global incidence. The age-standardized prevalence was 20.34 per 100,000, corresponding to approximately 270,719 cases, which remains lower than both the Asian and global averages. Nevertheless, epidemiological trends suggest that India has experienced a gradual rise in incidence over the past three decades, underscoring the growing public health significance of IBD in the region as well as globally (Giri et al., 2025). Beyond its prevalence, IBD is associated with a 1.5 to 5-fold higher mortality compared with the general population, with Crohn’s disease contributing most substantially to disease burden. Mortality is primarily driven by severe infections, progressive disease, surgical complications, and multiorgan involvement. Furthermore, patients, particularly those with pancolitis, carry a markedly elevated risk of colorectal cancer within two decades of disease onset, warranting regular colonoscopic surveillance every 1–2 years (Chung et al., 2018).

### 1.3 Aetiology and pathophysiology of inflammatory bowel disease

Although the precise aetiology of IBD remains elusive, it is widely accepted that its development involves a complex interplay of genetic susceptibility (genome), environmental exposures (exposome), gut microbiota dysbiosis, immune dysregulation, and epigenetic modifications. Key environmental factors encompassed within the exposome include air pollution, mode of delivery at birth, infant feeding practices (e.g., breastfeeding), dietary patterns, physical activity, socioeconomic status, geographic location, education level, infections, psychological stress, and tobacco use (Ananthakrishnan et al., 2018). More recently, microplastics (MPs) have emerged as a novel class of environmental pollutants of concern. Widespread in aquatic environments, they enter the body primarily through ingestion of food and water. Experimental studies suggest that MPs can impair intestinal barrier integrity, disrupt the gut microbiome, and promote metabolic disturbances, thereby contributing to IBD pathogenesis and worsening outcomes in individuals with preexisting gastrointestinal disorders (Ji et al., 2023).

**Fig. 1:**
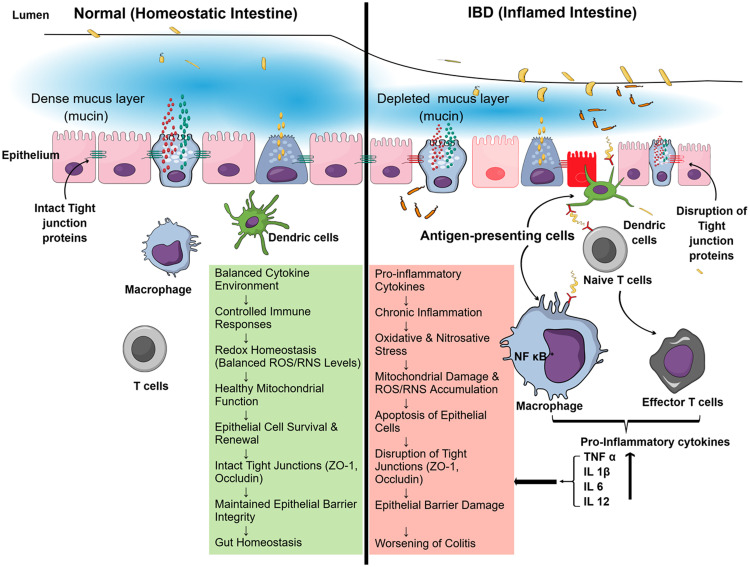
Pathophysiology of inflammatory bowel disease: the figure illustrates how tight junction defects, microbial imbalance, mucosal depletion, epithelial disruption, and immune cell activation (e.g., macrophages, dendritic cells, and T cells) contribute to the progression of inflammatory bowel diseases.

These factors converge to disturb the intestinal barrier, leading to the characteristic pathophysiology of IBD, especially ulcerative colitis. Epithelial injury or inflammation within the lamina propria undermines the integrity of the mucosal and mucin barriers, causing increased permeability and disruption of tight junctions (Kobayashi et al., 2020; Ordás et al., 2012). As a result, microbial antigens penetrate the mucosa and activate innate immune cells, including macrophages and dendritic cells, via toll-like receptors (TLRs). This activation triggers the NF-κB signalling pathway, driving the transcription of pro-inflammatory cytokines such as TNF-α, IL-1β, IL-6, IL-12, and IL-23, which together amplify and sustain the inflammatory cascade (Abraham et al., 2017; Tatiya-aphiradee et al., 2018) (Fig. 1).

Simultaneously, oxidative stress, characterised by excessive reactive oxygen species (ROS), further exacerbates tissue damage by disrupting signal transduction, inducing DNA damage, and triggering pro-inflammatory responses. ROS impair mucosal barrier function and stimulate macrophage activation, thereby reinforcing the inflammatory process (Muro et al., 2024). Key molecular pathways, including NF-κB, Nrf2, JAK-STAT, and BAX/BCL2, orchestrate the nexus between inflammation, oxidative stress, and cell survival, making them promising targets for therapeutic intervention in IBD.

### 1.4 Current therapeutic strategies and limitations

The management of IBD largely relies on pharmacological treatments. Standard therapies include aminosalicylates (e.g., sulfasalazine and other 5-aminosalicylic acid derivatives), corticosteroids (oral and rectal), and immunomodulators like thiopurines, methotrexate, calcineurin inhibitors, and Janus kinase (JAK) inhibitors. Biologic agents that neutralise pro-inflammatory cytokines, such as anti-TNF, anti-IL-12/23, and anti-integrin therapies, have significantly improved clinical outcomes. Other approaches, such as apheresis, target leukocyte reduction, while fecal microbiota transplantation (FMT) aims to restore microbial homeostasis. Stem cell-based therapies, including hematopoietic stem cells (HSCs), mesenchymal stem cells (MSCs), intestinal stem cells (ISCs), and exosome-based interventions, are also under investigation for IBD treatment (Cai et al., 2021).

For distal UC, glucocorticoids and 5-ASA derivatives such as mesalazine are often administered rectally, showing efficacy in mild to moderate disease. Corticosteroids, such as methylprednisolone, betamethasone, and hydrocortisone remain effective options (Ardizzone and Porro, 1998). In addition, non-conventional therapies have been explored, such as *Trichuris suis* ova therapy, which achieved 43% remission compared to 16.7% in placebo-treated patients (Summers et al., 2005). Recent biologic therapies include TNF-α inhibitors such as certolizumab, etanercept, and adalimumab. Other agents, including natalizumab, visilizumab, fontolizumab, alicaforsen, basiliximab, and antibodies targeting IL-12/IL-6, have also been investigated in steroid-refractory UC, although their long-term effectiveness in maintaining remission remains inconclusive (Meier and Sturm, 2011).

Many of these therapeutic strategies are also applicable to Crohn’s disease, although the treatment paradigm differs in emphasis and goals. Management of Crohn’s disease increasingly follows a treat-to-target (T2T) approach, emphasizing early intervention, tight disease control, and scheduled assessments of disease activity against predefined targets, such as clinical remission, normalization of biomarkers, and endoscopic healing. Therapies such as corticosteroids, exclusive enteral nutrition, anti-TNF agents, Janus kinase inhibitors (JAK-I), and interleukin-12/23 inhibitors generally induce clinical improvement within two months. In contrast, methotrexate, thiopurines, and vedolizumab may require longer to achieve maximal response. Therapy is escalated or modified if targets are not met, with non-invasive biomarkers and imaging increasingly employed to guide treatment decisions (Srinivasan, 2024)

Despite these therapeutic advancements, significant limitations remain due to adverse effects. Thiopurines may cause infections, myelosuppression, hepatotoxicity, pancreatitis, and an increased risk of hepatosplenic T-cell lymphoma, particularly when used in combination with anti-TNF agents. Methotrexate is associated with bone marrow suppression and organ toxicity, and is contraindicated in pregnancy owing to teratogenic risks. Anti-TNF agents carry risks of autoimmunity, infections, demyelinating conditions, heart failure, and malignancy. Notably, natalizumab has been linked to progressive multifocal leukoencephalopathy (PML) (McLean and Cross, 2014). Long-term therapy also carries additional risks, including skin cancer with thiopurines and increased susceptibility to tuberculosis, fungal infections, psoriasis, and melanoma with anti-TNF agents (Li et al., 2020c; Nardone et al., 2014; Solovan and Chiticariu, 2013; Xie et al., 2014). Importantly, these safety concerns are broadly shared between UC and CD therapies, although differences exist in response times, therapeutic endpoints, and overall treatment emphasis between the two diseases.

Given these drawbacks, there is an urgent need for therapies that extend beyond broad immunosuppression and account for patient heterogeneity. While current drugs already target inflammation and immune dysregulation, their effectiveness is often limited by off-target effects and variability in immune landscapes. Personalised medicine, tailored to genetic, microbial, and clinical profiles, offers a promising approach to improve efficacy and safety, with emerging strategies such as JAK/SMAD7 inhibitors, stem cell–based therapies, and systems biology–guided interventions showing potential (Ashton et al., 2019; Plichta et al., 2019). Importantly, future therapies should also address pathogenic features not adequately targeted by existing treatments, including gut microbiome dysbiosis, epithelial barrier dysfunction, and fibrosis, thereby moving toward a more comprehensive and durable paradigm for managing ulcerative colitis and Crohn’s disease (Costa et al., 2022).

### 1.5 Rationale for mechanistic approaches and non-animal models in IBD research

The successful development of targeted therapies for IBD depends on a more comprehensive mechanistic understanding of the disease’s complex biology. While conventional animal models have been instrumental in advancing our knowledge, they often fail to fully capture the complexity, heterogeneity, and dynamic nature of human IBD.

One of the central features of IBD pathogenesis is dysbiosis, characterised by marked alterations in the composition and function of the gut microbiota; however, the precise causal relationship remains unclear. In healthy individuals, the gut microbiota plays a crucial role in key physiological processes, including nutrient absorption, immune modulation, and maintaining epithelial barrier integrity. Among its metabolites, short-chain fatty acids (SCFAs), produced through the fermentation of dietary fibre, are crucial for intestinal homeostasis. They fuel colonocytes, promote mucosal healing, and modulate immune responses, while reduced butyrate production is a hallmark of dysbiosis and has been consistently linked to active IBD. Imbalances in the gut microbiota, particularly in genetically predisposed individuals, can lead to pathological outcomes, as demonstrated by the fact that such animals rarely develop spontaneous colitis under germ-free conditions (Laserna-Mendieta et al., 2018; Mukhopadhya and Louis, 2025; Shan et al., 2022).

Beyond microbial differences, animal models are constrained by fundamental discrepancies in immune responses, genetic architecture, and environmental exposures. Despite the availability of mammalian (rats, mice, rabbits, monkeys, dogs) and non-mammalian (zebrafish, fruit flies, nematodes) models, none fully replicates human IBD, and dedicated models for UC or CD remain elusive (Wen et al., 2023). Mice and humans differ in their immune system development, activation, and antigen responses (Mestas and Hughes, 2004). Moreover, murine studies often fail to adequately reflect the genetic and environmental diversity of human populations, even when diet, microbiome, or environmental variables are manipulated. While transgenic knockouts and antibody depletion are widely used to study gene function, human IBD risk rarely involves the complete loss of a single gene or protein (Katsandegwaza et al., 2022). Preclinical murine studies also overlook variability in therapeutic responses due to genetic polymorphisms. Outbred mice may mitigate this, but they require large sample sizes, which raises ethical and economic concerns (Chia et al., 2005).

Additional limitations include strain, diet, housing, and vendor-dependent variation in microbiota, as well as reduced microbial diversity in germ-free or barrier-raised mice compared to humans. Anatomical and functional differences in the gastrointestinal tract, along with region-specific variations in microbial composition, further hinder translational relevance. Collectively, these challenges underscore the need for more predictive, human-relevant models to investigate microbiota-driven mechanisms in IBD (Turner, 2018).

Ethical concerns surrounding the use of animals reinforce this need. Non-animal models such as 2D cultures, 3D organoids, and gut-on-chip systems provide controlled environments for study host–microbe interactions, aligning with the 3Rs principle (Replacement, Reduction, Refinement) (Bertorello et al., 2024). Complementing these, *in silico* approaches offer a cost-effective and environmentally sustainable means to simulate complex biological interactions, integrating data from both *in vivo* and *in vitro* studies (Bertorello et al., 2024). However, their success depends on rigorous experimental validation and ongoing refinement to ensure predictive accuracy.

Therefore, this review aims to present and evaluate the potential of next-generation non-animal models, including *in vitro* and *in silico* approaches, as promising alternatives to traditional animal models for advancing mechanistic understanding and therapeutic discovery in IBD.

## 2 *In vitro* NAMs in IBD research

### 2.1 2D models

#### 2.1.1 Caco-2 cells: a differentiated enterocyte-like model for intestinal barrier and cytokine modulation in IBD

Caco-2 cells, or cancer-coli-2 cells, are immortalised human colorectal adenocarcinoma cells initially established by Fogh in 1977 at the Sloan-Kettering Institute for Cancer Research (Caco-2 Cell Line, 2015; Fogh et al., 1977). When cultured, Caco-2 cells spontaneously differentiate into a heterogeneous population resembling intestinal epithelial cells (Caco-2 Cell Line, 2015). Upon differentiation, they exhibit several characteristics of mature small intestinal enterocytes, including the presence of apical brush-border microvilli and the expression of key digestive enzymes such as sucrase-isomaltase, dipeptidyl peptidase IV, lactase, aminopeptidase N, and peptidases (Lozoya-Agullo et al., 2017; Primavera et al., 2018).

**Fig. 2:**
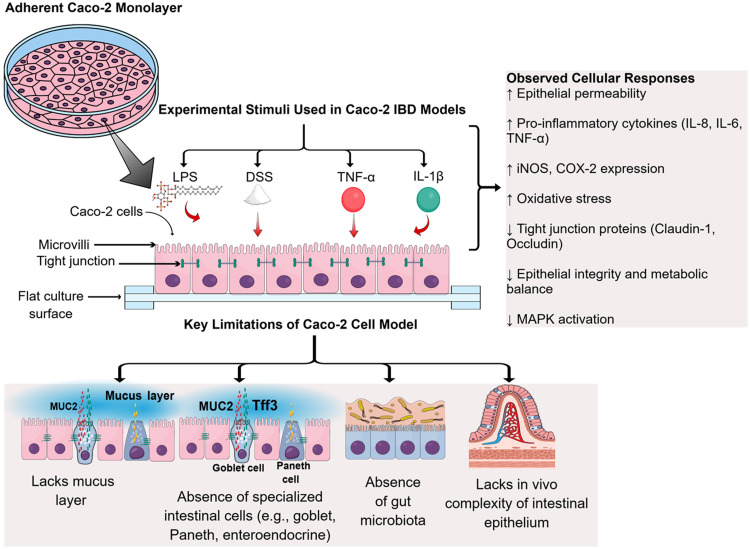
Caco-2 cells as a model for intestinal inflammation and barrier dysfunction; strengths and limitations.

Moreover, Caco-2 cells are capable of producing a range of pro- and anti-inflammatory cytokines, including interleukin (IL)-6, IL-8, IL-10, IL-15, IL-18, IL-23, tumor necrosis factor-α (TNF-α), and thymic stromal lymphopoietin (TSLP), thereby making them a valuable tool in inflammation-related research, particularly in the context of inflammatory bowel disease (IBD) (Borgonetti et al., 2022; Nanlohy et al., 2024). Due to their phenotypic and functional similarities to intestinal epithelial cells, Caco-2 cells have become a widely accepted *in vitro* model for studying intestinal physiology, drug absorption, and pathophysiological processes such as barrier dysfunction and inflammation (Fig. 2).

Despite their advantages, Caco-2 cells have certain limitations. The native intestinal epithelium is composed of multiple cell types, such as goblet cells, Paneth cells, and enteroendocrine cells, which are absent in monocultured Caco-2 systems. Moreover, *in vitro* cultures lack physiological features like the mucus layer and the unstirred water layer, both of which influence the solubility and absorption of compounds *in vivo*. The presence of bile acids and phospholipids also significantly affects the transport of lipophilic molecules, further limiting the translational relevance of *in vitro* findings (Caco-2 Cell Line, 2015). Nevertheless, due to their reproducibility and defined characteristics, Caco-2 cells remain suitable for prescreening anti-inflammatory interventions that target epithelial cytokine responses.

Several studies have demonstrated the utility of the Caco-2 cell model in identifying compounds that preserve intestinal barrier integrity and modulate inflammation. For example, rhamnogalacturonan (RGal), a polysaccharide isolated from *Acmella oleracea* leaves, has been shown to reduce IL-1β-induced epithelial permeability, suppress IL-8 secretion, and preserve tight junction proteins like claudin-1 and occludin in Caco-2 cells, thus suggesting its potential in maintaining intestinal barrier function (Maria-Ferreira et al., 2018). Similarly, a co-culture model using Caco-2 and RAW 264.7 cells revealed that activation of Exchange Protein Directly Activated by cAMP 2 (Epac-2) significantly mitigated lipopolysaccharide (LPS)-induced epithelial permeability and inflammation, highlighting its role in stabilising barrier function and regulating immune–epithelial interactions in conditions like Crohn’s disease (Song et al., 2009).

Furthermore, a study employing a dextran sulfate sodium (DSS)-induced Caco-2 model to mimic early-stage colitis evaluated the effects of bee pollen derived from *Camellia sinensis* L. (BP-Cs). Pretreatment with BP-Cs extract effectively countered DSS-induced cytotoxicity, oxidative stress, inflammation, and barrier disruption. It also inhibited MAPK signalling activation and preserved key metabolic pathways, particularly glycerophospholipid metabolism, underscoring its role in protecting epithelial integrity during the initial phases of colitis (Li et al., 2019). In another investigation, Liang et al. reported that corn protein hydrolysate digests significantly suppressed TNF-α-induced iNOS and COX-2 protein expression and reduced IL-8 production in Caco-2 cells, further supporting their utility in mechanistic anti-inflammatory research (Liang et al., 2020).

Together, these findings confirm the relevance of Caco-2 cell-based systems for studying epithelial inflammatory responses and for the initial screening of therapeutic or nutraceutical candidates intended to alleviate intestinal barrier dysfunction in inflammatory disorders, such as IBD.

#### 2.1.2 HT-29 cells: A dynamic model for mucosal inflammation, goblet cell function, and cytokine-mediated pathways

HT29 cell line is derived from human colon cancer. In 1964, Fogh and Trempe derived it from a 44-year-old Caucasian woman’s primary tumour (New Human Tumor Cell Lines, 1975). These cells drew attention due to their ability to express mature intestinal cell features such as enterocytes or mucus-generating cells, i.e., goblet cells (HT29 Cell Line, 2015). Additionally, HT29 cells can undergo various patterns of differentiation depending on modifications to the culture medium or the addition of differentiation-inducing agents. HT-29 cells, because their differentiation can be modulated, are particularly appropriate for observing the onset of structural and molecular events leading to cell polarity (Use of Cultured Cell Lines in Studies of Intestinal Cell Differentiation and Function, 1991). HT29 cells also release digestive enzymes comparable to Caco-2 cell lines (Use of Cultured Cell Lines in Studies of Intestinal Cell Differentiation and Function, 1991).

Previous studies have demonstrated that HT-29 cells, similar to Caco-2 cells, are capable of releasing cytokines, making them a suitable *in vitro* model for screening phytomedicines. For instance, Qingchang Suppository Powder (QCSP) has been shown to inhibit the IL-17A signalling pathway in HT-29 cells, suggesting a novel mechanism by which QCSP may exert its therapeutic effects against UC (Zhou et al., 2021). An additional advantage is observed in the HT29-MTX cell line, a methotrexate-treated derivative of HT-29 cells, which produces mucin at relatively high levels. This characteristic more closely mimics the native intestinal mucosal environment, thereby enhancing their utility in gastrointestinal research (Adamczak et al., 2016).

HT-29 cell lines are widely utilised in colitis research due to their relevance in modelling intestinal epithelial responses. Numerous studies have demonstrated their utility in evaluating the efficacy and mechanisms of various therapeutic agents. For instance, berberine has been shown to protect HT-29 cells from TNF-α-induced epithelial barrier disruption by preserving E-cadherin expression, reducing permeability, and downregulating the adhesion molecules ICAM-1 and CD62E. It also inhibited the production of pro-inflammatory chemokines IL-8 and IP-10, and blocked THP-1 immune cell adhesion, highlighting its role in maintaining barrier integrity and modulating IEC–immune interactions (Li et al., 2020b).

In cancer-related applications, curcumin significantly inhibited the proliferation of HT-29 colon cancer cells and reduced COX-2 expression. When combined with 5-fluorouracil (5-FU), it exerted a synergistic anti-proliferative effect, leading to a nearly six-fold reduction in COX-2 protein levels, suggesting enhanced therapeutic efficacy through COX-2 inhibition (Du et al., 2006). Similarly, sodium butyrate (NaB) induced notable enterocytic differentiation and growth inhibition in HT-29 cells. Although 1,25-dihydroxyvitamin D₃ [1,25-(OH)₂D₃] alone had a limited impact, its combination with NaB significantly enhanced goblet cell maturation, alkaline phosphatase activity, and overall differentiation, indicating a synergistic differentiation-promoting effect (Tanaka et al., 1989).

Further supporting the utility of HT-29 cells in phytochemical research, Rajendiran et al. evaluated the effects of *Alpinia officinarum* extract, which led to dose-dependent cytotoxicity, reduced cell viability, and downregulation of NF-κB and COX-2 expression (Rajendiran et al., 2018). In another study, researchers demonstrated that goniothalamin exerted pro-apoptotic effects on HT-29 cells via ROS generation, MAPK activation, and caspase-dependent apoptosis (Vendramini-Costa et al., 2016).

In the context of ulcerative colitis, Xiaotian et al. explored the effects of p-hydroxybenzaldehyde, a compound derived from the cyanobacterium *Nostoc commune*, and found that it enhanced mRNA expression of inflammatory mediators in LPS-stimulated HT-29 cells, supporting its potential role in IBD modulation (Xu et al., 2021). Additionally, HT-29 cells have been widely employed in toll-like receptor (TLR) studies to investigate innate immune mechanisms in IBD pathogenesis, reflecting their growing importance in TLR-related signalling research (Damoogh et al., 2021).

Taken together, the ability of HT-29 cells to mimic key aspects of the colonic epithelium, particularly mucosal inflammation, cytokine responses, and goblet cell differentiation, establishes them as a versatile and physiologically relevant *in vitro* model for mechanistic exploration and therapeutic screening in IBD research.

#### 2.1.3 T84 cells: a polarised colonic epithelium model for tight junction integrity and inflammatory signalling in IBD

The T84 cell line, originally established in 1980 by Murakami and Masui from a human colon carcinoma xenograft in nude mice, has emerged as a widely utilised *in vitro* model for studying intestinal physiology and pathophysiology, particularly in the context of hormonal regulation of colonic epithelial cells (Murakami and Masui, 1980). These cells grow as polarised monolayers with well-developed tight junctions (TJs) and apical microvilli, closely mimicking the architecture of native human colonic epithelium (Dharmsathaphorn et al., 1984). Unlike Caco-2 cells, which undergo spontaneous differentiation, T84 cells require external induction, such as with transforming growth factor-β1 (TGF-β1) or soluble mesenchymal factors, to differentiate into crypt-like epithelial structures (Madara and Dharmsathaphorn, 1985; Ponce De León-Rodríguez et al., 2019). This feature provides a controlled system to investigate epithelial maturation and region-specific intestinal functions.

Functionally, T84 cells are instrumental in exploring electrolyte transport mechanisms (Alzamora et al., 2011; Beltrán et al., 2015; Tang et al., 2010), intestinal permeability (Huang et al., 2016; Jennis et al., 2018), and most notably, inflammatory signalling pathways associated with intestinal disorders (Cho and Park, 2015; Wongkrasant et al., 2020). Due to their ability to form physiologically relevant epithelial barriers, they are frequently employed in host microbiota interaction studies (Devriese et al., 2017; Otte and Podolsky, 2004), making them a valuable model for deciphering mucosal immune responses and epithelial integrity.

Recent advances have further underscored the relevance of T84 cells in inflammatory bowel disease (IBD) research. One study demonstrated that lipopolysaccharide (LPS) exposure induced phosphorylation of claudin proteins, potentially contributing to barrier dysfunction (Li et al., 2015). Another investigation reported that treatment with a commercial energy drink formulation significantly suppressed the secretion of pro-inflammatory cytokines IL-6 and TNF-α, indicating its potential impact on epithelial immune signalling (Manzini et al., 2021). In addition, high-throughput cDNA microarray analysis has been employed to investigate the regulatory roles of long non-coding RNAs (lncRNAs) in ulcerative colitis using the T84 model (Chen et al., 2021). These findings collectively support the use of T84 cells in mechanistic studies focused on inflammation, barrier modulation, and gene regulation in IBD contexts.

Despite their utility, T84 cells, like other colorectal carcinoma-derived lines such as Caco-2 and HT-29, exhibit inherent limitations due to their malignant origin. These include altered gene expression profiles and a lack of fully differentiated epithelial functionality seen in *in vivo* tissues, which may affect the translational relevance of experimental outcomes (Rahman et al., 2021). Overall, the T84 cell line offers a robust and physiologically relevant platform for investigating intestinal barrier function, immune responses, and molecular signalling in IBD. Its ability to form polarised monolayers with functional tight junctions, respond to inflammatory cues, and undergo induced differentiation makes it a valuable model for dissecting epithelial dynamics and evaluating therapeutic interventions in colitis research.

### 2.2 3D models & organoids

#### 2.2.1 3D model in IBD

Three-dimensional (3D) cell culture models have emerged as powerful tools in biomedical research, providing a more physiologically relevant alternative to conventional two-dimensional (2D) monolayer cultures. By better mimicking cellular architecture, heterogeneity, and the native tissue microenvironment, 3D systems reduce dependence on animal models and enhance translational relevance (Cacciamali et al., 2022). These models utilise advanced bioengineering techniques and scaffolding systems to reconstruct complex tissue-like structures, making them particularly valuable for studying disease mechanisms, evaluating drug responses, and exploring applications in regenerative and precision medicine.

A 3D cell culture is defined as “a cell culture that can mimic a living organ’s organisation and microarchitecture (Huh et al., 2011). Advances in bioengineering have transformed traditional cell culture into a dynamic and innovative assay, enabling the study of tissue and organ behaviour under *in vitro* conditions. As highlighted by researchers, the inclusion of a third dimension effectively bridges the gap between basic cell culture and living tissue, significantly enhancing the utility of cell-based assays (Pampaloni et al., 2007). A critical milestone in the development of 3D models is the recognition of the importance of cellular context, particularly as it relates to key processes such as proliferation, migration, and apoptosis (Bissell et al., 2003).

This innovative approach helps bridge the gap between *in vitro* and *in vivo* models by integrating novel biotechnologies, such as hydrogels that closely mimic the extracellular matrix (ECM) and modulate growth factor activity. The ECM plays a pivotal role in delivering biochemical and mechanical signals that influence cellular behaviour and fate (Nikolova and Chavali, 2019). Its composition can vary widely in physical and chemical properties. Collagen is commonly employed to facilitate cell adhesion in gels like polyacrylamide. Hydrogels are another critical component in 3D systems; modifications in their composition have been explored to study how matrix properties influence mesenchymal stem cell spreading, proliferation, and differentiation (Chaudhuri et al., 2016).

In recent years, 3D models have gained increasing attention for their superiority over 2D systems, particularly in recapitulating essential cellular features such as morphology, migration patterns, and gene expression. Furthermore, the use of human cells in 3D cultures minimises interspecies variation and enhances the translational value of experimental findings, allowing a more accurate representation of human pathophysiology. This is especially important in inflammatory bowel disease (IBD) research, where 3D systems have been effectively utilised to study intestinal inflammation, barrier dysfunction, and immune interactions.

**Fig. 3:**
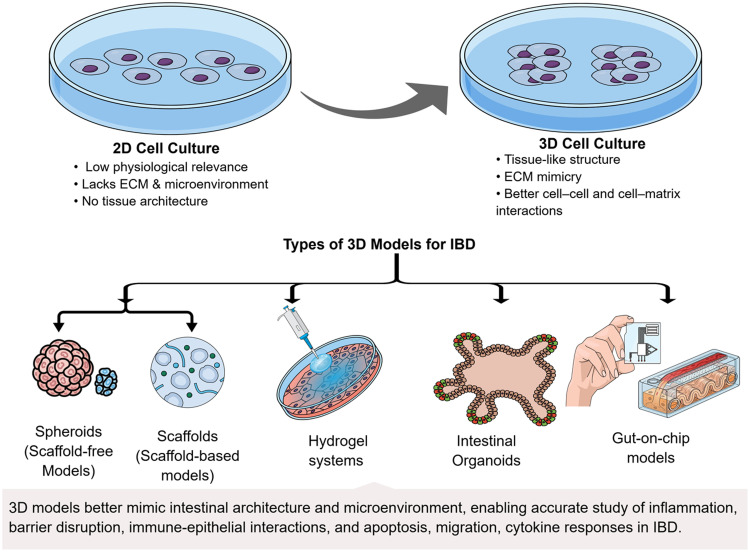
3D cell culture models for IBD: bridging the gap between *in vitro* and *in vivo*.

Several 3D intestinal inflammation models have been developed that partially replicate IBD features. These include scaffold-based models, hydrogel systems, and decellularised tissue constructs, along with more advanced platforms such as organoids and intestine-on-a-chip technologies. These approaches continue to evolve, offering increasingly sophisticated models that capture the complexity of IBD. The major types of 3D models and their respective strengths and limitations in modelling the IBD inflammatory microenvironment are summarised in Table 1 and further elaborated in the following sections (Fig. 3).

**Table 1:** Summary of various 3D models used to simulate IBD: strengths and limitations in replicating an inflammatory microenvironment (Andrews and Kriegstein, 2022; Ferreira et al., 2024; Srivastava et al., 2024).

3D Model	Strengths	Limitations
**Spheroid, Hydrogel, and Scaffold-Based 3D Cultures**	Relatively reproducible and simple; supports cell–cell and cell–ECM interactions; functional in drug screening, stem cell, and immunotherapy studies	No vasculature, leading to poor nutrient/oxygen delivery and necrotic core formation; low reproducibility; heterogeneous size; imaging/analysis challenges
**Organ-on-a-Chip**	Mimics organ-level functions; integrates flow and mechanical stimuli; allows real-time, high-resolution analysis; improves human translatability	Lack of standardized fabrication and universal medium; technically complex and costly; limited immune simulation; challenges in scalability and reproducing physiologically relevant conditions
**Organoids**	Derived from pluripotent and adult stem cells, these cells mimic early tissue architecture, crypt–villus organization, and key organ-level functions (absorption, secretion, barrier integrity, cytokine responsiveness, and epithelial regeneration); patient-specific; allow investigation of atypical cellular, molecular, and genetic features; valuable for drug screening, regenerative medicine, epithelial repair, and potential cell replacement.	Low reproducibility; lack of vasculature; limited maturation; atypical physiology; poor immune integration; lack of arealization

#### 2.2.2 Co-culture systems of intestinal systems

Co-culture-based 3D intestinal models have evolved from simple monolayer systems on permeable supports into more complex platforms that better mimic native tissue architecture, enabling improved studies of drug transport, metabolism, and disease modelling, especially where mucus production plays a critical role (Helena Macedo et al., 2021).

Co-culture systems significantly enhance the physiological relevance of intestinal models, particularly when immune cells are incorporated to replicate the inflammatory environment more accurately. Leonard et al. developed a 3D co-culture model using Caco-2 cells alongside immunocompetent macrophages and dendritic cells to mimic the inflamed intestinal mucosa (Leonard et al., 2010). In this setup, controlled inflammation was induced using proinflammatory stimuli such as IL-1β, IFN-γ, and LPS, with IL-1β showing the strongest effect. The model exhibited a moderate upregulation of inflammatory markers and a 20% reduction in transepithelial electrical resistance (TEER), indicating compromised barrier integrity. Significantly, the inclusion of immune cells amplified cytokine responses and enhanced nanoparticle uptake, demonstrating the model's potential for evaluating drug formulations, xenobiotic interactions, and therapeutic strategies targeting IBD-associated inflammation (Leonard et al., 2010).

Building on this, a team of researchers refined the 3D co-culture model by incorporating standardised immune cell lines, macrophage-like THP-1 and dendritic-like MUTZ-3 cells, into the collagen scaffold to improve reproducibility and facilitate cytotoxicity assessments (Susewind et al., 2015). This model, combined with Caco-2 cells, effectively simulated the intestinal mucosa under both healthy and inflamed conditions. Upon IL-1β-induced inflammation, the model exhibited heightened IL-8 secretion and preserved epithelial integrity, closely mimicking an inflamed intestinal barrier. When tested with engineered nanoparticles (TiO₂, Ag, and Au), silver nanoparticles showed the highest toxicity, though interestingly, Caco-2 monocultures were more susceptible than the 3D co-cultures. These findings highlight the enhanced physiological relevance and predictive capacity of this advanced 3D co-culture model, particularly for evaluating nanoparticle toxicity in inflammatory settings relevant to IBD (Susewind et al., 2015).

To further enhance physiological relevance, another group of researchers developed a sophisticated triple co-culture model using a silk protein-based tubular scaffold incorporating Caco-2/HT29 epithelial cells and primary human intestinal fibroblasts (H-InMyoFibs) (Chen et al., 2015). This biomimetic construct supported mucus production (10–20 μm thick), promoted epithelial differentiation, and enabled compartmentalised tissue organisation. The model recreated key features of the native intestine, including luminal oxygen gradients, mucus-secreting surfaces, and host-microbial interaction zones. Notably, the inward-facing epithelial monolayer and underlying fibroblasts established oxygen tension gradients similar to those observed *in vivo*, making the model suitable for investigating both physiological processes and disease states such as chronic intestinal inflammation or microbial dysbiosis. While limitations in long-term functionality were noted, integration with dynamic perfusion systems holds promise for extended application. This scalable and highly tunable system represents a significant advancement for studying host-microbe interactions, drug delivery, and the role of hypoxia in intestinal pathophysiology (Chen et al., 2015).

Building on earlier co-culture models, a group of scientists further refined the intestinal 3D system by incorporating an inflammatory component to better replicate the pathophysiological environment of IBD (Dosh et al., 2019). They cultured Caco-2 and HT29-MTX cells (9:1) on thermo-responsive L-pNIPAM hydrogel scaffolds under dynamic conditions, achieving long-term co-cultures maintained for 6 and 11 weeks. This configuration fostered the development of finger-like villus structures and supported differentiation markers such as alkaline phosphatase (ALP) and mucin (MUC2) production. The model was subjected to pro-inflammatory cytokines (IL-1β and TNFα) and hypoxia (1% O₂) to simulate inflamed intestinal environments. These stimuli resulted in notable downregulation of the tight junction protein ZO-1, reflecting increased epithelial permeability, a hallmark of IBD. MUC2 expression, in contrast, was upregulated in response to cytokines and hypoxia, underscoring mucosal responses to inflammation. Furthermore, the system revealed differential expression of inflammation-associated enzymes, including MMP2, MMP9, and ADAMTS1, validating its potential to model complex cellular dynamics under disease-like conditions. This study demonstrated the value of L-pNIPAM hydrogel scaffolds for sustaining long-term epithelial co-cultures and highlighted the importance of incorporating inflammatory cues to emulate key features of IBD pathology. However, the reliance on tumour-derived cell lines remains a limitation, and future work should explore integration with primary or stem-cell-derived organoids to enhance physiological fidelity (Dosh et al., 2019).

Another notable advancement involved the development of a simplified co-culture system using Caco-2 epithelial cells and PMA-differentiated THP-1 macrophages to replicate both healthy and inflamed intestinal states. Under baseline conditions, the model-maintained barrier integrity without triggering inflammatory or cytotoxic responses. However, upon stimulation with IFN-γ and LPS to mimic inflammation, the system exhibited hallmark features of intestinal injury, including transient barrier disruption and elevated levels of pro-inflammatory cytokines and cytotoxicity markers. This dual-state model offers a practical platform for evaluating immune responses and testing therapeutic interventions in the context of intestinal inflammation (Kämpfer et al., 2017).

Building on this, another study introduced a 3D tissue model incorporating non-transformed human colon organoid-derived epithelial cells and a subepithelial layer of monocyte-derived macrophages within a biomaterial scaffold (Roh et al., 2019). This model successfully recapitulated key features of intestinal architecture, including microvilli, mucus production, and immune cell infiltration. Upon inflammatory stimulation with LPS and IFN-γ, the system exhibited hallmark morphological and cytokine responses reflective of active IBD, such as epithelial disruption and upregulation of IL-1β, IL-6, CXCL10, MCP-2, and MIP-1β. This bioengineered platform offers a physiologically relevant tool for investigating immune-epithelial interactions and screening IBD therapies (Roh et al., 2019).

Finally, a “leaky gut” co-culture model combining Caco-2, THP-1, and MUTZ-3 cells has been developed to emulate the severely compromised epithelial barrier characteristic of IBD (Hartwig et al., 2022). This model, showing sub-confluent epithelial integrity and heightened immune responsiveness, enabled assessment of permeability and target engagement of anti-inflammatory agents, including tofacitinib and JAK1-targeted siRNA nanomedicine. Notably, the model facilitated quantification of JAK/STAT signalling inhibition and nanoparticle uptake within immune cells near damaged epithelial zones. This system offers a powerful *in vitro* platform to evaluate the performance of novel nanomedicines and targeted therapies under conditions that closely resemble inflamed intestinal mucosa in IBD (Hartwig et al., 2022), (Table 2).

**Table 2:** Summary of co-culture intestinal models for IBD research.

Co-Culture Model Description	Cell Types Used	Inflammatory Stimuli	Key Features / Applications	References
**3D Macrophage–Dendritic Cell Co-Culture**	Caco-2 + macrophages + dendritic cells	IL-1β, IFN-γ, LPS	20% TEER reduction; enhanced cytokine release; nanoparticle uptake	(Leonard et al., 2010)
**Collagen-Based 3D Immune Model**	Caco-2 + THP-1 + MUTZ-3	IL-1β	Increased IL-8, preserved integrity; nanoparticle toxicity tested (Ag > TiO₂/Au); better than monoculture	(Susewind et al., 2015)
**Silk Tubular Scaffold System**	Caco-2 + HT29 + *H*-InMyoFibs	Native oxygen gradient; microbial contact zones	Mucus (10–20 μm); epithelial differentiation; hypoxia zone; microbial studies	(Chen et al., 2015)
**L-pNIPAM Hydrogel Long-Term Model**	Caco-2 + HT29-MTX (9:1)	IL-1β, TNF-α, hypoxia (1% O₂)	Long-term viability (6–11 weeks); ZO-1 downregulation; MUC2 upregulation; MMPs expression	(Dosh et al., 2019)
**Simplified macrophage-epithelial co-culture**	Caco-2 + PMA-differentiated THP-1	IFN-γ, LPS	Dual-state model; cytokine elevation; transient barrier disruption	(Kämpfer et al., 2017)
**Colon Organoids with Monocyte-Derived Macrophages**	Non-transformed epithelial organoids + macrophages	LPS, IFN-γ	Mucus, microvilli, immune infiltration; IL-1β, IL-6, CXCL10, MCP-2, MIP-1β upregulation	(Roh et al., 2019)
**Leaky Gut Model (Reduced Epithelial Confluency)**	Caco-2 + THP-1 + MUTZ-3	IFN-γ (for JAK/STAT activation)	Low TEER; 92% confluency; siJAK1-nanoparticle accumulation and signaling inhibition	(Hartwig et al., 2022)

#### 2.2.3 Gut-on-chip technologies

To unravel the complexities of gut physiology and its associated diseases, researchers are turning to advanced *in vitro* platforms. One such innovation is the organ-on-a-chip technology, which has emerged as a cutting-edge alternative to traditional models. Utilising microfluidic systems, this platform replicates the three-dimensional architecture and dynamic physiological conditions of native gut tissue, including mechanical forces, luminal flow, and cellular interactions, thereby offering a more accurate and controllable environment for investigating intestinal functions (Valiei et al., 2023).

Importantly, gut-on-a-chip systems can be tailored using various parameters, including specific intestinal cell lines (e.g., Caco-2, HT-29, or organoid-derived epithelia), diverse microfluidic designs (mono- or multi-channel systems), and mechanical cues like peristalsis-mimicking motion. These customizable features enable the modelling of crucial gut processes such as epithelial barrier integrity, immune-microbiome interactions, and host-pathogen responses, making organ-on-a-chip a powerful and physiologically relevant tool for gastrointestinal research and therapeutic screening (Valiei et al., 2023).

Various gut-on-a-chip models have been developed to simulate the complex physiology of the intestine using microfluidic platforms. Early mono-environment designs focused on culturing a single cell type, such as Caco-2 cells, to mimic intestinal epithelium under dynamic fluid flow (Fois et al., 2021; Guo et al., 2018). More advanced multi-environment systems enabled compartmentalised cocultures, offering improved modelling of epithelial-microbial and immune interactions. For instance, the HMI device by Marzorati et al. (2014) physically separates microbial and epithelial cultures using a porous membrane and mucus layer, thus preserving cell viability while minimising cytotoxicity. Similarly, the HuMiX platform by Shah et al. introduced a third channel to simulate the basal side of the epithelium, enhancing tissue viability and microbial co-culture efficiency (Shah et al., 2016).

Further advancements came from Emulate’s commercial gut-on-a-chip, developed by Ingber’s group (Kim et al., 2012), which integrated dynamic mechanical strain and fluid flow to simulate peristalsis, enabling spontaneous formation of villi-like structures and mucin secretion (Kim et al., 2012; Kim and Ingber, 2013). Other innovations include collagen scaffold–supported villi topographies (Shim et al., 2017), convoluted microchannel layouts for fluid dynamics (Shin et al., 2020), and circular luminal geometries mimicking *in vivo* gut symmetry (Jing et al., 2020). These designs not only extend tissue viability but also enhance physiological relevance, making gut-on-a-chip platforms highly valuable for disease modelling and drug testing.

An illustrative example is a double-layered microfluidic chip mimicking the intestinal barrier by culturing Caco-2 cells with U937 immune cells under continuous apical and basolateral perfusion. This setup-maintained shear stress, tight junction integrity, and uniform growth. Upon LPS and TNF-α exposure, reduced TEER values indicated increased permeability, while suppressed cytokine expression highlighted the barrier’s protective immune function, underscoring its utility for studying host-microbe and immune interactions in a dynamic, *in vivo*-like environment (Ramadan and Jing, 2016).

Extending the utility of such platforms, a study employed a high-throughput 3D gut-on-a-chip model using the OrganoPlate® platform to replicate key features of IBD. Caco-2–like enterocytes were stimulated with IL-1β, TNF-α, and IFN-γ, resulting in barrier disruption and elevated secretion of pro-inflammatory cytokines such as IL-8, CCL20, and IP-10 markers associated with immune cell recruitment and inflammation. Treatment with the anti-inflammatory compound TPCA-1 restored barrier integrity and reduced cytokine levels in a dose-dependent manner. Additionally, adenoviral shRNA-mediated knockdown of RELA and MYD88 significantly attenuated the inflammatory response, highlighting the model’s utility for drug screening and target validation in IBD research (Beaurivage et al., 2019).

To further bridge the gap between *in vitro* systems and *in vivo* complexity, gut-on-a-chip models have also been adapted to investigate the role of microbiota in intestinal health. One such model co-cultured probiotics with epithelial cells under dynamic flow without using synthetic membranes. This configuration successfully supported cell polarisation, mucus production, and barrier integrity. Importantly, probiotic treatment restored function in injured epithelium, emphasising the model’s relevance for studying host–microbe interactions in IBD (Jeon et al., 2022). Building on this concept, a recent study proposed a simplified yet robust gut-on-a-chip platform that integrates both microbial interactions and physiologically relevant oxygen gradients, an often-overlooked feature of gut physiology. By fine-tuning microchannel dimensions, selectively blocking oxygen diffusion, and independently perfusing aerobic and hypoxic media, the model recreated steep oxygen gradients across the epithelium. Upon TNF-α and LPS exposure, an IBD-like inflammatory state was induced. Subsequent treatment with *Bifidobacterium bifidum* preserved epithelial barrier function and promoted monolayer repair, possibly through ZO-1 co-localisation. This low-cost, scalable strategy not only advances our understanding of microbiome-host crosstalk in IBD but also offers a practical platform for high-throughput screening and personalised medicine applications (Liu et al., 2023).

Together, these advances in gut-on-a-chip platforms ranging from immune-competent and high-throughput models to oxygen-gradient-integrated systems underscore their transformative potential in unravelling the complex pathophysiology of IBD. By faithfully recapitulating dynamic host–microbiome interactions, barrier functions, and inflammatory responses, these microfluidic models offer a physiologically relevant, scalable, and ethically viable alternative to animal testing, paving the way for precision medicine, drug discovery, and targeted therapeutic interventions in IBD.

#### 2.2.4 Human intestinal organoids

Over the past decade, human intestinal organoids (HIOs) have gained prominence as advanced *in vitro* systems for studying inflammation, enabling personalised drug testing and providing valuable insights into epithelial responses and microenvironmental dynamics following mucosal injury. As we know, the gastrointestinal tract is a complex organ that is constantly exposed to foreign materials and organisms. The intestinal epithelium is a single layer of cells, with entire cell turnover every 2–6 days (Williams et al., 2015). Intestinal stem cells, marked by Lgr5 expression, reside at the crypt base, shielded from the lumen and supported by niche signals from mesenchymal and Paneth cells. Wnt signalling, crucial for maintaining their stemness via Ascl2 activation, drives Lgr5⁺ cells to generate rapidly dividing transit-amplifying cells (TA), which later differentiate into specialised intestinal lineages (Schuijers et al., 2015). Unlike the small intestine, the colon lacks Paneth cells; instead, its stem cell niche is supported by signals from the underlying mesenchyme and a specialized epithelial population known as Deep Crypt Secretory (DCS) cells. DCS cells, located at the base of colonic crypts, share some similarities with Paneth and goblet cells but represent a distinct lineage characterized by Reg4 expression. In contrast to Paneth cells, DCS cells do not produce Wnt ligands; instead, they primarily express EGF and Notch ligands. In addition, DCS cells express immunoregulatory and host-defense genes, including *Retnlb*, underscoring their emerging roles in epithelial barrier integrity and mucosal homeostasis (Schumacher, 2023).

Intestinal organoids can be generated from two primary sources: tissue-resident intestinal stem/progenitor cells isolated from primary crypts, or induced pluripotent stem cells (iPSCs) that are first established and characterized as the starting population. In the tissue-derived approach, intestinal stem cells (ISCs), either as single Lgr5⁺ cells or within intact crypts, are isolated from donor tissue (typically human or mouse) using mechanical or enzymatic dissociation methods. These ISCs are then embedded into a supportive extracellular matrix, commonly Matrigel or similar laminin-rich hydrogels, to mimic the native stem cell niche (Kleinman and Martin, 2005; Zhao et al., 2022). Once seeded, the cells are maintained in a specialised culture medium supplemented with essential growth factors, including Wnt, R-spondin, Noggin, Jagged1, and EGF, which collectively support ISC proliferation, self-renewal, differentiation, and spatial organisation into three-dimensional organoids (Holmberg et al., 2017; Kleinman and Martin, 2005). Over time, these cultures develop into complex structures comprising multiple intestinal cell types, such as absorptive enterocytes, goblet cells, and enteroendocrine cells, closely resembling the architecture and cellular diversity of native intestinal tissue (Zhao et al., 2022). These organoids can be sustained *in vitro* for long durations, acting as miniature replicas of the donor intestine, often called "mini guts" or intestinal avatars (Jung et al., 2011). Their ability to mimic patient-specific intestinal tissue supports their use in personalised therapy, disease modelling, and studies of IBD mechanisms. As reliable *ex vivo* models, they have become valuable tools for investigating intestinal diseases and developing tailored treatment approaches (Günther et al., 2022).

Several research studies have investigated the use of human intestinal organoids (HIOs) to model inflammation, particularly within the context of inflammatory bowel disease (IBD). These investigations have addressed diverse aspects of disease pathology, including the modelling of epithelial inflammatory injury (Xing et al., 2023), bacterial–host interactions (Study Bacteria–Host Interactions Using Intestinal Organoids, 2016), and the monitoring of IBD-associated epigenetic alterations (Ghorbaninejad et al., 2023). Additionally, HIOs have been employed to simulate inflammatory environments through cytokine stimulation (Kandilogiannakis et al., 2021), elucidate key molecular pathways involved in IBD pathogenesis (Boye et al., 2022), and uncover cytokine-responsive gene regulatory networks that drive mucosal inflammation (Pavlidis et al., 2022).

Let’s see a few HIOs and their application in IBD. A notable example involves the generation of an IBD organoid model using DSS to induce inflammation. These organoids exhibited hallmark features of the intestinal epithelium, with expression of markers such as LGR5, BMI1, MUC2, and E-cadherin, closely resembling those observed in stained human intestinal tissues. Upon DSS treatment, there was a marked upregulation of pro-inflammatory cytokines and their downstream effector genes, resulting in disrupted epithelial integrity and increased paracellular permeability. This model effectively recapitulates key aspects of mucosal inflammation and barrier dysfunction, making it a valuable platform for evaluating therapeutic strategies in IBD research (Rallabandi et al., 2020). Complementing this approach, Woznicki et al. established a cytokine-induced IBD-like organoid model to study epithelial cell death pathways. They demonstrated that IFN-γ and TNF-α synergistically trigger intestinal epithelial cell death via a non-canonical CASP8-JAK1/2-STAT1 axis, independent of classical apoptosis or necroptosis pathways. Validated in Crohn’s disease-derived organoids, this model is now instrumental in screening JAK inhibitors and caspase-targeted therapies aimed at restoring barrier function and mitigating inflammation (Woznicki et al., 2021). Another study using enteroid organoids (EnO) derived from Crohn’s disease (CD) patients and the SAMP1/YitFc mouse strain (SAMP), which mimics intestinal epithelial barrier dysfunction (IEBD), demonstrated reduced expression of intestinal stem cell (ISC) markers and Wingless-related integration site 3A (Wnt3a) targets. This suggests an impairment of the Wnt pathway. Additionally, the transcriptomic and protein analysis of CD-derived EnOs confirmed altered metabolic profiles, defective unfolded protein response (UPR), and abnormal secretory lineages. These findings further underscore the importance of organoid-based approaches in modelling disease-specific epithelial alterations in IBD (Buttó et al., 2020).

Extending this line of evidence, IBD patient-derived colon organoid cultures revealed persistent epithelial abnormalities, even in the absence of immune cell stimulation. These defects were likely due to chronic exposure to inflammatory mediators or underlying epigenetic modifications. Interestingly, organoids derived from non-ulcerated yet adjacent inflamed zones retained disease-associated features such as altered epithelial polarity, tight junction disruption, and aberrant gene expression (e.g., MUC2, LYZ, CLDN18). Upon stimulation with an inflammatory cytokine cocktail, control organoids mimicked several pathological features observed in IBD-derived organoids, highlighting the long-term imprinting potential of inflammatory cues. Moreover, the study demonstrated that pharmacological agents such as anti-TNF, corticosteroids, and 5-ASA partially restored epithelial integrity in a dose-dependent manner. This supports the potential of organoid models as personalised platforms for evaluating patient-specific therapeutic responses and unravelling epithelial dysfunction in IBD (d’Aldebert et al., 2020).

Personalised medicine applications are equally promising, as demonstrated by a study in which patient-derived enteroids and colonoids from pediatric Crohn’s disease (CD) patients retained disease-specific transcriptomic signatures even when derived from macroscopically unaffected rectal tissue. Inflammatory pathways such as cytokine–cytokine receptor interaction and chemokine signalling remained upregulated, and cytokine stimulation *in vitro* reactivated disease-associated genes. This highlights the potential of organoid models to reflect patient-specific epithelial alterations, making them valuable tools for translational IBD research (Karakasheva et al., 2023).

Taken together, these findings establish human intestinal organoids (HIOs) as powerful and physiologically relevant platforms for studying intestinal inflammation, barrier dysfunction, and epithelial regeneration in IBD. By faithfully recapitulating patient-specific molecular signatures, structural features, and functional responses to inflammatory stimuli, HIOs bridge the gap between *in vitro* experimentation and clinical relevance. Their ability to model complex host–microbe interactions, epigenetic memory, cytokine signalling networks, and therapeutic responsiveness underscores their pivotal role in mechanistic investigations and personalised medicine approaches for IBD. As such, organoid-based systems are not only transforming our understanding of intestinal pathophysiology but also accelerating the development of targeted, patient-tailored interventions.

Advancing this field, the integration of human intestinal organoids with gut-on-chip platforms signifies a pivotal advancement in the development of next-generation *in vitro* models. Multiple integrative methodologies have already illustrated how this hybrid strategy can overcome the constraints of static organoid cultures. For example, the wrapping of perfusable vasculature around organoids in iFlow plates enabled modeling of immune cell recruitment during colon inflammation (Rajasekar et al., 2020), whereas perfusion devices with duodenum organoids recreated villi-like structures, intestinal folds, and peristaltic motion, supported by transcriptomic profiles closely resembling the native intestine (Kasendra et al., 2018). Similarly, colon-on-chip systems incorporating mucus bilayers reproduced the thickness and goblet cell composition of the human colon (Sontheimer-Phelps et al., 2020), while crypt-like hydrogel topographies with luminal perfusion enabled the study of radiation injury and chronic parasite infection (Nikolaev et al., 2020). Despite these advances, major issues remain, particularly the absence of integrated gastrointestinal smooth muscle cells necessary for replicating peristalsis and the insufficient inclusion of other relevant cell types, such as mesenchymal, neural, and immune cells (Zhao et al., 2024). Therefore, the continuous refinement of integrated organoid-on-chip platforms will be pivotal for faithfully capturing epithelial–immune–microbiota interactions, thereby shaping the future of advanced *in vitro* models for IBD research and enabling the development of precision therapeutics.

## 3 *In silico* NAMs in IBD research

*In silico* approaches refer to the use of computer-based simulations and computational models to study biological processes, offering a powerful alternative to traditional *in vivo* and *in vitro* methods. Within the context of IBD research, *in silico* NAMs are increasingly employed to accelerate drug discovery, predict toxicity, and explore disease mechanisms. These techniques facilitate virtual screening of compound libraries, rational drug design based on protein–ligand interactions, and simulation of disease pathways using large-scale data sets. The ability to perform high-throughput, multidimensional analyses enables researchers to uncover molecular targets, assess potential adverse effects, and prioritise candidate therapeutics with increased efficiency and reduced reliance on animal models. *In silico* NAMs thus play a pivotal role in enhancing translational relevance, refining risk assessment, and supporting the development of personalised interventions in IBD (In Silico Methods, 2014; Mesbah-Uddin et al., 2015).

The application of *in silico* NAMs in IBD research encompasses a diverse array of computational strategies, each contributing uniquely to the understanding of disease mechanisms and therapeutic discovery. Key approaches include molecular docking and dynamics simulations for target interaction prediction, AI/ML-based models for compound screening and toxicity forecasting, network pharmacology for mapping multi-target pathways, and toxicogenomics supported by integrated databases such as LINCS, CTD, and ToxCast.

### 3.1 Molecular docking & dynamics

Molecular docking and molecular dynamics (MD) are complementary *in silico* techniques widely used in drug discovery and mechanistic studies. Docking predicts the optimal orientation and binding affinity of small molecules to target proteins using scoring functions based on van der Waals forces, electrostatics, and shape complementarity. It enables high-throughput virtual screening and facilitates understanding of protein–ligand and protein–protein interactions. Docking methods are generally classified as receptor-based, which utilise the 3D structure of the target protein, or ligand-based, which relies on known inhibitors. Standard tools include AutoDock, DOCK, FTDOCK, HEX, ArgusLab, and CHARMM. MD simulations extend docking by modelling the atomic motion of molecules over time under physiological conditions, revealing conformational dynamics, binding stability, and energetics. Together, these techniques are crucial for refining drug candidates and mapping signalling pathways, particularly in inflammatory diseases like IBD. Typically, IBD drug discovery begins with identifying disease-relevant targets based on their mechanistic role and expression levels, followed by biological validation, *in silico* screening, and preclinical evaluation for pharmacokinetics, toxicity, and efficacy (Danese et al., 2016; Hemant U Chikhale, 2020; Wankhede et al., 2024).

This integrated approach has shown significant utility in IBD research, where it has been employed to identify and evaluate small molecules targeting key inflammatory mediators. Notably, several studies have demonstrated successful docking and simulation of compounds with critical regulatory proteins such as NF-κB, Nrf2, and components of the JAK/STAT pathway, underscoring their potential as therapeutic targets for modulating intestinal inflammation (Guo et al., 2024; Lv et al., 2024; Touny et al., 2025). For example, a molecular docking study explored the interaction of polyphenolic compounds from *Ocimum tenuiflorum* with Keap1, a key negative regulator of Nrf2. The results demonstrated that several ligands effectively bound to the Kelch domain of Keap1, potentially disrupting the Keap1–Nrf2 interaction and promoting Nrf2 nuclear translocation. Among these, catechin showed the strongest binding affinity, with a minimum binding energy of −8.2 kcal/mol, indicating its potential as a potent Keap1 inhibitor. These findings highlight catechin’s promise as a therapeutic candidate for IBD management through Nrf2 pathway modulation, though further *in vitro* and *in vivo* validation is warranted (Kumar and Sarkar, 2023). Similarly, TNF-α, a central mediator in IBD-related inflammation, has been targeted in a virtual screening study involving FDA-approved drugs. Using molecular docking, ADMET profiling, and molecular dynamics simulations, iopromide and deferoxamine emerged as promising candidates with strong binding affinities to TNF-α (PDB ID: 2AZ5). These compounds demonstrated favorable interaction profiles and stability, suggesting their potential for drug repurposing in IBD therapy (Halder et al., 2023).

Collectively, these studies exemplify how *in silico* approaches, integrating docking, dynamics, and pharmacological profiling, can accelerate the discovery and optimisation of novel therapeutic agents for IBD. This progress is grounded in the growing identification of molecular targets that reflect the complex and multifactorial nature of IBD pathogenesis. Several key targets have been elucidated across different biological pathways, including integrins such as ITGA4 and ITGB7, which mediate leukocyte trafficking and are often upregulated in IBD, making them prime candidates for inhibition (Ochoa et al., 2021). Cytokine signalling components, including IL-12B, TNF, JAK2, JAK3, and TYK2, are also heavily implicated, particularly through their involvement in the pro-inflammatory JAK-STAT cascade (Dubinsky et al., 2013; Ochoa et al., 2021). Enzymes like cyclooxygenases (PTGS1/2) and ALOX5, responsible for prostaglandin and leukotriene synthesis, respectively, are similarly targeted to curb inflammatory responses (Fain, 1987; Ochoa et al., 2021). In addition, matrix metalloproteinases (MMP1, MMP7, MMP13), which contribute to extracellular matrix degradation and tissue injury, are inhibited to preserve mucosal integrity (Meyer et al., 2021; Ochoa et al., 2021; Vandenbroucke et al., 2013). On the other hand, nuclear receptors such as PPARγ, NR3C1, and VDR, often found to be downregulated in IBD, are targeted for activation due to their critical roles in modulating inflammation and maintaining epithelial homeostasis (Dubuquoy, 2006; Schote et al., 2019). Other emerging targets include DHFR (purine synthesis), S1PR1 (lymphocyte trafficking), ATP4A (acid secretion), and PPAT (nucleotide biosynthesis), many of which are upregulated and offer further opportunities for therapeutic intervention. Together, these diverse molecular targets provide a strong foundation for rational drug design using computational strategies, enabling the development of more precise and effective IBD therapies (Johnson et al., 2023).

In conclusion, molecular docking serves as a powerful and cost-effective tool in drug design, enabling the identification and optimization of therapeutic candidates by targeting key molecular pathways involved in IBD. When integrated with molecular dynamics and *in silico* ADMET profiling, it not only accelerates the discovery of novel or repurposed drugs but also reduces the reliance on animal studies during the early stages of drug development. By allowing high-throughput virtual screening and mechanistic insight into protein–ligand interactions, this approach offers a reliable pre-screening platform that can refine and prioritise compounds for further *in vitro* validation, thereby minimising unnecessary animal use in the preclinical pipeline.

### 3.2 Artificial intelligence /machine learning *in silico* models for diagnosis and prognosis in IBD

Artificial intelligence (AI), an interdisciplinary field rooted in computer science, engineering, and cognitive sciences, aims to replicate aspects of human intelligence through machines. Introduced by John McCarthy in 1965, AI has evolved to include machine learning (ML), a subfield that enables systems to learn from data and make predictions without being explicitly programmed. ML encompasses various approaches, including supervised learning (e.g., support vector machines, random forests), unsupervised learning (e.g., clustering algorithms), and deep learning, a more advanced technique employing neural networks for high-dimensional data analysis (Panch et al., 2018; Wang and Siau, 2019). These methods have recently gained traction in inflammatory bowel disease (IBD) research, offering promising tools for diagnosis, patient stratification, disease severity assessment, and prediction of therapeutic outcomes.

In the diagnostic landscape of IBD, ML has demonstrated the ability to enhance disease classification. Mossotto et al. (2017) evaluated 287 pediatric IBD patients and showed that integrating endoscopic and histological features significantly improved the classification accuracy of disease subtypes. Supervised models reached up to 82.7% accuracy, and this performance rose to 83.3% in an independent validation cohort. Interestingly, unsupervised models identified four novel patient clusters, highlighting the strength of ML in both categorising and uncovering previously unrecognised disease patterns (Mossotto et al., 2017). Rubin et al. (2019) applied the CITRUS supervised learning algorithm to mass cytometry data from blood and intestinal biopsies of 68 IBD patients. They identified an eight-marker immune signature that discriminated Crohn’s disease (CD) from ulcerative colitis (UC), achieving an AUC of 0.845, supporting the use of blood-based immune profiles for IBD subtype classification (Rubin et al., 2019). Romagnoni et al. (2019) employed penalised logistic regression, gradient-boosted trees, and artificial neural networks on Immunochip data from over 18,000 CD patients and 34,000 controls. All models demonstrated comparable performance (AUC = 0.80), validating the capacity of ML to stratify genetic risk and reveal both established and novel SNP associations with CD (Romagnoni et al., 2019).

In assessing disease severity, convolutional neural networks (CNNs) have been particularly effective in analysing endoscopic images. Klang et al. developed a CNN trained on 17,640 capsule endoscopy images to detect small-bowel ulcers in CD patients. The model achieved excellent diagnostic performance, with AUCs ranging from 0.94 to 0.99 at both image and patient levels, emphasising the potential of deep learning to support endoscopic evaluations (Klang et al., 2020). Similarly, Barash et al. constructed an ordinal CNN trained on 17,640 images to grade CD ulcers according to the PillCam CD classification automatically. The model achieved classification accuracies of 0.91 (grade 1 vs. 3), 0.78 (grade 2 vs. 3), and 0.624 (grade 1 vs. 2), demonstrating promise in standardised ulcer severity grading (Barash et al., 2021). Bhambhvani et al. developed a deep CNN to automate Mayo Endoscopic Score (MES) grading in UC using 777 colonoscopy images. The model showed intense discrimination among MES 1, 2, and 3, with AUCs of 0.96, 0.86, and 0.89, respectively, and an overall accuracy of 77.2%, suggesting the model's usefulness in disease monitoring and therapy assessment (Bhambhvani and Zamora, 2021).

AI has also been applied to predict therapeutic responses and clinical outcomes in IBD. Popa et al. created a neural network model incorporating baseline clinical and biological data to predict endoscopic disease activity in UC patients undergoing anti-TNF therapy. The model achieved 90% accuracy and an AUC of 0.92 in the test set, and remarkably reached 100% accuracy (AUC = 1.0) in the validation cohort, showing its potential to guide biologic therapy decisions (Popa et al., 2020). Sofo et al. applied a support vector machine (SVM) algorithm to preoperative electronic health record (EHR) data from 32 UC patients undergoing total abdominal colectomy. The model predicted minor postoperative complications with an accuracy of 84.3%, sensitivity of 87.5%, and specificity of 83.3%, underlining the feasibility of AI for pre-surgical risk stratification using routinely available clinical parameters (Sofo et al., 2020).

The application of AI and ML in IBD has expanded rapidly, providing powerful *in silico* tools to analyse and interpret complex datasets, ranging from genetic and molecular signatures to histological images, endoscopic data, and electronic health records (Gubatan et al., 2021). Although their use remains largely within the research domain, ongoing advances in algorithmic precision and data integration promise to transition these tools into clinical settings. Importantly, such *in silico* approaches offer the ability to perform rapid pre-screening, risk stratification, and virtual testing, which can expedite early diagnosis and therapeutic decision-making. By enabling high-throughput, non-invasive analyses, these technologies may significantly reduce dependence on traditional *in vivo* and *in vitro* experiments, supporting more ethical, efficient, and personalised IBD care. Furthermore, future studies must explore their potential in areas such as colorectal cancer surveillance within IBD populations to fully harness their clinical utility.

### 3.3 Network pharmacology

Network pharmacology integrates bioinformatics, network biology, and polypharmacology to overcome the limitations of the traditional one-drug/one-target paradigm by elucidating complex interactions between bioactive compounds and disease-associated genes. This systems-based approach is particularly valuable for multi-component therapies like those used in Ayurveda and traditional Chinese medicine (TCM), providing a scientific framework to uncover their mechanisms and therapeutic potential (Li et al., 2023b; Network Pharmacology, 2017).

In a study by Kong et al., network pharmacology combined with molecular docking was employed to identify the active components of licorice targeting ulcerative colitis (UC), supported by a TCM-component–target–disease network model. Active compounds were screened using the TCMSP, PubChem, and SwissTargetPrediction databases, while UC-related disease targets were retrieved from GeneCards and OMIM. The overlapping targets between licorice components and UC were visualised using Cytoscape, and core targets were identified through topological analysis of the protein–protein interaction (PPI) network generated via STRING, highlighting key nodes in the therapeutic network (Kong et al., 2024). Similarly, Wu et al. combined microarray analysis, network pharmacology, and molecular docking to investigate the mechanism of Huangqin decoction (HQD) in ulcerative colitis. Active compounds and targets were identified using TCMSP, DrugBank, and SwissTargetPrediction, while UC-related targets were obtained from GEO (GSE107499). Venn analysis revealed 79 overlapping targets, and PPI network analysis identified core genes including IL6, TNF, IL1B, PTGS2, ESR1, and PPARG. GO and KEGG pathway enrichment highlighted the TNF and IL-17 signalling pathways. Molecular docking showed strong binding of HQD components quercetin, baicalein, and wogonin to core targets, suggesting a multi-target, multi-pathway therapeutic mechanism (Wu et al., 2022). In a related study, Liu et al. employed network pharmacology integrated with experimental validation to elucidate the pharmacological mechanisms of Pulsatilla decoction (PD) in Crohn’s disease (CD). By analyzing bioactive compounds and disease-associated targets using drug–compound–target–disease networks, PPI analysis, and GO/KEGG enrichment, they identified 134 intersecting targets, with HSP90AA1 emerging as a key node. Enrichment analysis pointed to antifibrotic pathways involving AKT, mTOR, ERK1/2, and PKC signalling. Experimental validation in CD patient tissues and a CD fibrosis mouse model confirmed that PD treatment reduced fibrosis and downregulated these signalling targets, supporting the multi-target, multi-pathway therapeutic potential of PD in CD management (Liu et al., 2022b). Similarly, You et al. applied a network pharmacology-based strategy to investigate the mechanisms of TongXieYaoFang (TXYF) in inflammatory bowel disease (IBD). Through database mining, target prediction, enrichment analysis, and molecular docking, 34 overlapping targets were identified between TXYF and IBD, with key pathways involving NF-κB and Toll-like receptor signalling. Docking studies showed strong binding of TXYF compounds to PTGS2, highlighting its anti-inflammatory potential and supporting its traditional use in IBD management (You et al., 2022).

Beyond traditional formulations, network pharmacology has also been applied to modern therapeutics, enabling novel target identification, efficacy prediction, and drug repurposing in IBD. By integrating genomic, transcriptomic, and pharmacological datasets, it supports the precision use of biologics and small molecules (Johnson et al., 2023). For example, a cross-cohort study in ulcerative colitis mapped transcriptomic data onto the Human Interactome to derive a molecular signature predicting inadequate response to infliximab, achieving high predictive accuracy (AUC = 0.83) and stratifying responders from non-responders (Ghiassian et al., 2022). Similarly, network medicine approaches in Crohn’s disease and ulcerative colitis have expanded disease gene modules and identified candidate repurposable drugs, underscoring their translational value for modern IBD therapeutics (Kitani et al., 2021).

These studies collectively demonstrate that network pharmacology serves as a powerful *in silico* approach for elucidating the complex mechanisms of multi-component therapies in inflammatory bowel diseases. By integrating chemical, genomic, and pharmacological data, it enables the identification of active compounds, key targets, and signalling pathways involved in disease modulation. The combination of bioinformatics tools, network analysis, and molecular docking not only validates traditional formulations like licorice, HQD, PD, and TXYF but also paves the way for evidence-based integration of traditional medicine into modern therapeutic strategies for IBD and related disorders.

### 3.4 Toxicogenomics & databases

Toxicogenomics, which integrates toxicology with genomics, plays a crucial role in identifying gene expression changes and molecular pathways associated with drug toxicity in IBD drug development (Suter et al., 2004). By leveraging large-scale genomic databases such as GEO, CTD, and DrugBank, researchers can systematically evaluate the safety profiles of candidate small molecules, predict off-target effects, and uncover gene–environment interactions relevant to intestinal inflammation. These databases, combined with *in silico* modelling and virtual screening, enhance the precision and efficiency of identifying safe, targeted therapies, making toxicogenomics an essential component of computational strategies in modern IBD drug discovery (Johnson et al., 2023).

To support toxicogenomic-based drug discovery in IBD, several bioinformatics databases and tools are available that enhance the accuracy of target identification, interaction prediction, and safety profiling. For instance, the Open Targets Platform provides a systematic framework for identifying and prioritising therapeutic targets based on diverse evidence types (Ochoa et al., 2021), while DrugBank offers detailed annotations on drug mechanisms and known targets (Wishart et al., 2008). Tools like SELF-BLM (Keum and Nam, 2017), iDTIESBoost (Rayhan et al., 2017), and NetCBP (Chen and Zhang, 2013) apply machine learning and network-based approaches to predict drug–target interactions, even in cases lacking prior interaction data. The GEO database serves as a valuable resource for transcriptomic data, aiding in functional genomics and biomarker discovery (The Gene Expression Omnibus Database, 2016). Additionally, DisGeNET (Piñero et al., 2021) and the Comparative Toxicogenomics Database (CTD) (Davis et al., 2021) integrate gene-disease and chemical-gene relationships, enabling the assessment of environmental and drug-related impacts on disease pathways. Databases such as PDTD (Gao et al., 2008), TTD (Chen, 2002), and UniProt (The UniProt Consortium et al., 2021) Further contribute by providing curated protein targets, therapeutic links, and protein function data. Together, these tools create a robust computational ecosystem that accelerates *in silico* drug development and improves the identification of safe and effective therapeutic candidates for IBD.

Therefore, *in silico* approaches such as toxicogenomics and specialised bioinformatics databases present a transformative pathway for advancing drug discovery in IBD. These methods enable the rapid identification of disease-associated genes, prediction of drug–target interactions, and early assessment of safety profiles, significantly reducing the time and cost associated with traditional drug development. By leveraging computational tools for genomic analysis, target prioritisation, and virtual screening, researchers can decode the complex molecular networks underlying IBD and design precise, multi-targeted small-molecule therapies. Embracing these technologies holds great promise for delivering more effective, personalised, and safer treatments for IBD in the evolving landscape of modern medicine.

## 4 Modelling emerging pathways in IBD via NAMs

**Fig. 4:**
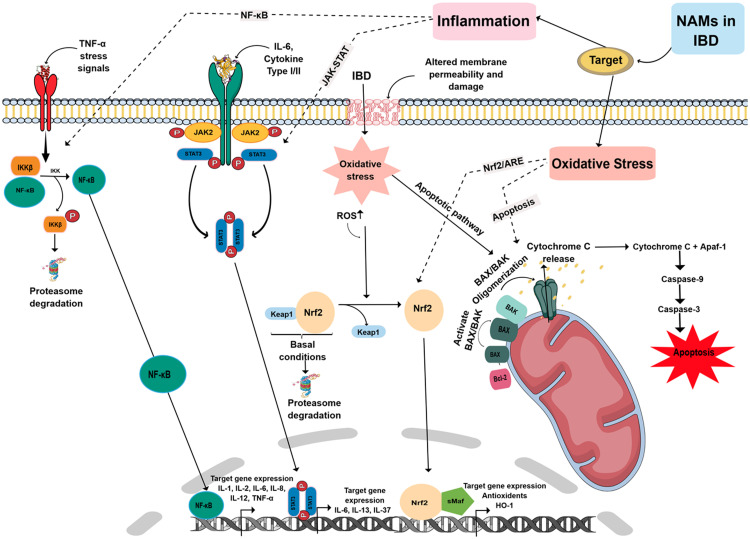
NAMs target NF-κB, JAK-STAT, Nrf2, and apoptosis in IBD: a multi-pathway approach to restore gut homeostasis.

Inflammatory bowel diseases (IBD) are complex, multifactorial conditions involving several interconnected signalling pathways that regulate oxidative stress, immune responses, and intestinal epithelial barrier function. Among these, the nuclear factor erythroid 2-related factor 2 (Nrf2)/antioxidant response element (ARE) pathway plays a critical role in preserving redox homeostasis and protecting against oxidative stress-induced damage (Li et al., 2023a; Piotrowska et al., 2021). Concurrently, the nuclear factor kappa B (NF-κB) pathway acts as a central regulator of inflammation by promoting the transcription of multiple pro-inflammatory mediators (156). Another central axis, the Janus kinase/signal transducer and activator of transcription (JAK-STAT) pathway, governs cytokine-mediated immune and inflammatory responses essential to IBD pathogenesis (Chen et al., 2024; Salas et al., 2020; Sarapultsev et al., 2023) (Fig. 4).

Environmental triggers, such as heavy metal exposure, further exacerbate IBD through metal-induced oxidative stress, which amplifies inflammation and intestinal injury (Narvey et al., 2025). Intestinal barrier dysfunction, marked by impaired mucosal integrity and increased epithelial permeability, is another key contributor to disease initiation and persistence (Zhao et al., 2025). These overlapping mechanisms highlight the dynamic interplay among oxidative stress, immune dysregulation, and barrier disruption in the chronic inflammation seen in IBD.

Therapeutically, targeting both classical and emerging pathways, including Nrf2/ARE, NF-κB, JAK-STAT, metal-induced stress responses, and barrier dysfunction, offers promising avenues for intervention. A focus on specific molecular nodes within these signalling networks facilitates a comprehensive understanding of disease mechanisms and the identification of druggable targets. In this regard, integrative approaches using new approach methodologies (NAMs), such as molecular docking and network pharmacology, are increasingly being used in early-stage drug discovery.

NF-κB remains a prime target due to its pivotal role in inflammation. It regulates genes encoding key cytokines (IL-1, IL-2, IL-6, IL-8, IL-12, TNF-α), chemokines (IL-18, MCP-1), and inflammasome components (NLRP3, pro-IL-1β) (162). A docking study demonstrated that daidzein (DZ) and equol (EQ) effectively bind to the P65–P50 dimer interface (∼−5.4 kcal/mol). DZ forms hydrogen bonds with Cys197 (P65), Thr304, Arg308, and Lys278 (P50), while EQ binds Cys197 and Arg198 (P65), and His307 and Lys278 (P50), indicating potential to inhibit NF-κB activation (162). Additionally, a network pharmacology and docking study on Gegen Qinlian Decoction (GQD) identified NF-κB as a central target among 59 hub genes relevant to ulcerative colitis. Baicalein and berberine, key GQD constituents, showed strong affinity for NF-κB, supporting their role in mitigating NF-κB-mediated inflammation (Liu et al., 2021).

Similarly, the JAK-STAT pathway is another vital inflammatory axis. Cytokine-induced activation of JAKs results in the phosphorylation and nuclear translocation of STAT, leading to the transcription of immune-regulatory genes (Salas et al., 2020; Sarapultsev et al., 2023). Although monoclonal antibodies targeting specific cytokines have improved outcomes, their effectiveness is often limited by primary or secondary non-response (Salas et al., 2020). Small-molecule JAK inhibitors like tofacitinib, approved for ulcerative colitis, offer broader action by targeting multiple cytokine pathways simultaneously (Liu et al., 2022a). Next-generation JAK inhibitors are currently under advanced clinical investigation, with promising safety and efficacy profiles.

The Nrf2/ARE pathway serves as a key defence against reactive oxygen species (ROS), maintaining epithelial homeostasis and modulating inflammation (Piotrowska et al., 2021). Given the multifaceted pathology of IBD, a combination approach using Nrf2 activators with NF-κB or JAK-STAT inhibitors is gaining attention as a multi-targeted therapeutic strategy. Natural products and probiotics that activate Nrf2 are being explored for their safety and multifunctionality (Li et al., 2023a). Supporting this, a study by Kumar et al. used molecular docking to investigate interactions between Keap1 (a negative regulator of Nrf2) and polyphenols from *Ocimum tenuiflorum* (Krishna Tulsi). Catechin showed the highest binding affinity (−8.2 kcal/mol), forming hydrogen bonds with GLY364, LEU365, and LEU557, suggesting its potential to disrupt Keap1-Nrf2 binding and enhance Nrf2 activity (Kumar and Sarkar, 2023).

Essential heavy metals such as copper (Cu), iron (Fe), zinc (Zn), manganese (Mn), chromium (Cr [III]), cobalt (Co), selenium (Se), and nickel (Ni) are indispensable for enzymatic processes, antioxidant activity, and immune regulation. However, their dysregulation, either through deficiency or overload, is increasingly implicated in IBD. In contrast, nonessential metals like arsenic (As), aluminium (Al), cadmium (Cd), chromium(VI), lead (Pb), mercury (Hg), and titanium (Ti) have no physiological role and they are toxic even in trace amounts (Narvey et al., 2025). Metal toxicity can compromise barrier integrity, induce dysbiosis, and trigger immune responses via oxidative and inflammatory pathways (Narvey et al., 2025; Turner, 2006). For example, arsenic exposure induces small intestinal toxicity in mice by disrupting barrier function and activating RhoA/ROCK and TLR4/MyD88/NF-κB signaling pathways (Zhao et al., 2023). Chronic Cr and/or Ni exposure leads to intestinal toxicity characterized by increased IL-6, IL-1β, IL-18, TNF-α, and IFN-γ, activation of NF-κB and NLRP3 inflammasome pathways, and reduced IL-10 (Gu et al., 2024). Similarly, aluminum promotes NLRP3 inflammasome activation, inducing IL-1β and IL-17α, while chromium enhances NF-κB and NLRP3 signaling, elevating pro-inflammatory cytokines and suppressing IL-10. Lead suppresses Nos2/Cox2 and NF-κB–mediated cytokine production, but upregulates MTs and Hmox1, and alters gut microbiota composition. Mercury activates the ASK1/JNK apoptotic pathway with caspase-3 involvement, whereas titanium influences tight junction proteins (ZO-1, occludin) but shows inconsistent inflammatory outcomes (Narvey et al., 2025), (Table 3).

**Table 3:** Overview of the effects of heavy metals and micro-/nanoplastics on intestinal barrier, microbiome, immunity, and associated signaling pathways.

Substance	Effects (Barrier, Microbiome, Immune)	Pathways Involved
**Arsenic (As)**	Disrupts barrier integrity, induces small intestinal toxicity, promotes inflammation	RhoA/ROCK, TLR4/MyD88/NF-κB
**Chromium (Cr) & Nickel (Ni)**	Increase IL-6, IL-1β, IL-18, TNF-α, IFN-γ; activate inflammasome; reduce IL-10; barrier toxicity	NF-κB, NLRP3 inflammasome
**Aluminum (Al)**	Activates inflammasome, increases IL-1β and IL-17α	NLRP3 inflammasome
**Lead (Pb)**	Alters cytokine production, upregulates MTs and Hmox1; shifts microbiota	NF-κB suppression; MT/Hmox1 induction
**Mercury (Hg)**	Damages epithelial cells, alters microbiota, activates apoptosis via caspase-3	ASK1/JNK pathway
**Titanium (Ti)**	Alters tight junction proteins (ZO-1, occludin); inconsistent inflammation	Barrier-related (TJ proteins)
**Copper (Cu)**	Excess disrupt gut microbiota and mucosal integrity; deficiency impairs repair; immune dysregulation	NF-κB, ROS stress
**Polystyrene MPs/NPs (PS-MPs)**	Cause barrier leakage, ↓ TJ proteins, ↑ pro-inflammatory mediators	TLR4/MyD88/NF-κB, ROS/NLRP3/MLCK
**Polyethylene MPs (PE-MPs)**	Induce dysbiosis, Th17/Treg imbalance, inflammation	TLR4/AP-1/IRF5
**Polyvinyl chloride MPs (PVC-MPs)**	↓ Mucus (Muc1, Muc2, Klf4); alter microbiota & bile acids; ↑ permeability & inflammation	Microbiota–bile acid–SCFA axis; metabolic pathways (ABC transporters, bile secretion, vitamin absorption, pyrimidine metabolism, steroid biosynthesis)

**Note:** NF-κB, Nuclear Factor kappa B; TLR4, Toll-like Receptor 4; MyD88, Myeloid Differentiation Primary Response 88; NLRP3, NOD-, LRR- and pyrin domain-containing protein 3; RhoA/ROCK, Ras homolog family member A/Rho-associated protein kinase; IL, Interleukin; TNF-α, Tumor Necrosis Factor-alpha; IFN-γ, Interferon-gamma; MTs, Metallothioneins; Hmox1, Heme oxygenase 1; ASK1, Apoptosis Signal-regulating Kinase 1; JNK, c-Jun N-terminal Kinase; TJ, Tight Junction; MLCK, Myosin Light Chain Kinase; AP-1, Activator Protein 1; IRF5, Interferon Regulatory Factor 5; SCFA, Short-Chain Fatty Acids; ABC, ATP-Binding Cassette.

In addition to heavy metals, micro- and nanoplastics (MNPs) are emerging as important environmental triggers, since ingestion is a major exposure route. Studies suggest that MNPs can disrupt the gut barrier, alter the gut microbiome, and contribute to intestinal inflammation (Agrawal et al., 2024). For instance, exposure to polystyrene microplastics (PS-MPs) has been shown to induce colonic inflammation and intestinal barrier leakage by activating the TLR4/MyD88/NF-κB signaling pathway, accompanied by reduced expression of tight junction proteins and elevated pro-inflammatory mediators, thereby linking MNP exposure to gut barrier dysfunction and IBD-like pathology (Wang et al., 2025). Similarly, PS-MPs can trigger intestinal toxicity by inducing oxidative stress and activating the ROS-dependent NF-κB/NLRP3/MLCK signaling pathway, which downregulates tight junction proteins and promotes barrier dysfunction, thereby exacerbating colonic inflammation (Zeng et al., 2024). Polyethylene microplastics (PE-MPs) have also been shown to disrupt intestinal homeostasis by inducing gut dysbiosis and triggering inflammation through the TLR4/AP-1/IRF5 signaling pathway, along with altered Th17/Treg cell balance and elevated pro-inflammatory cytokines (Li et al., 2020a). Polyvinyl chloride microplastics (PVC-MPs) further impair mucus secretion (↓ Muc1, Muc2, Klf4), alter gut microbiota and metabolite profiles, disrupt bile acid metabolism, and reduce the abundance of butyrate-producing bacteria, such as Faecalibacterium. This cascade drives epithelial permeability and inflammation via the microbiota–bile acid–SCFA axis (Chen et al., 2022) (Table 3).

While MNPs represent a novel class of environmental pollutants that influence gut inflammation, classical dietary factors such as essential mineral deficiencies also play a critical role in IBD pathology. Calcium deficiency is linked to osteoporosis in IBD patients (Caplan et al., 2017), while reduced serum magnesium correlates with higher disease activity (Trapani et al., 2018). Copper deficiency, particularly in those with short bowel syndrome, is associated with anaemia, fractures, mood disorders, and vascular complications (Kanikowska et al., 2015; Moon et al., 2021). Notably, inflammation may elevate serum copper levels, potentially masking an underlying deficiency (Prohaska, 2014).

Among essential metals, copper best exemplifies the paradox of metal homeostasis in IBD. While necessary for enzymatic and immune functions, excess copper exerts antimicrobial and pro-oxidative effects that disrupt gut microbiota and mucosal integrity. IBD-associated dysbiosis, marked by *Enterobacteriaceae* overgrowth such as *Escherichia coli*, may involve bacterial adaptation to resist host-mediated metal stress. Macrophages, in turn, deploy copper at sites of inflammation as an antimicrobial strategy, inadvertently contributing to local tissue damage (Braymer and Giedroc, 2014; Sheldon and Skaar, 2019).

Given the complexity of IBD, encompassing oxidative stress, immune dysregulation, metal imbalance, barrier dysfunction, and emerging environmental triggers such as microplastics, traditional animal models often fall short in replicating the human-specific pathophysiology. In contrast, NAMs such as *in silico* modelling, molecular docking, network pharmacology, and toxicogenomics offer human-relevant, high-throughput alternatives. These tools can predict metal–protein interactions, simulate key signalling pathways (e.g., NF-κB, Nrf2, JAK/STAT), and assess compound efficacy across multiple targets with greater precision. By integrating omics data and minimising species-specific differences, NAMs provide a more accurate and ethical platform for exploring disease mechanisms and accelerating the development of targeted IBD therapies.

## 5 Challenges and future perspectives

Although non-animal models such as cell cultures and computational platforms are promising, they come with notable limitations. Cell culture methodologies, particularly when used for modelling intestinal physiology and inflammation, often require specialised equipment, sterile handling, and extensive care, which can be both costly and time-consuming (Vinyard and Faciola, 2022). However, these investments are considered worthwhile due to their potential to explore cellular mechanisms previously inaccessible through traditional *in vivo* models. Moreover, the successful implementation of these systems depends heavily on well-trained personnel, reinforcing the need for workforce development in NAM-based research. While immortalised cell lines offer practical advantages in terms of availability and ease of culture, they harbour oncogenic traits that may not accurately reflect normal physiological responses. Conversely, non-immortal (primary) cell cultures, although more biologically relevant, are limited by poor viability and high cell death during isolation and propagation (Kent-Dennis et al., 2020; Zhan et al., 2017). This highlights the need for methodological refinement to improve both efficiency and reproducibility in *in-vitro* IBD models.

On the *in silico* front, platforms such as molecular docking and network pharmacology provide high-throughput and cost-effective insights but lack critical components such as immune system complexity and gut microbiome integration, thereby limiting their translational applicability (Sahu et al., 2024). Additionally, current scoring functions and conformational sampling algorithms often vary across different software platforms, leading to inconsistencies and reduced predictive power. Integration of multi-omics datasets, patient-derived genomic profiles, and machine learning algorithms may offer improved biological relevance and precision. Nonetheless, widespread regulatory acceptance remains a major hurdle due to the lack of standardised benchmarks and validated pipelines. Future directions should prioritise the development of interdisciplinary frameworks that bridge computational, experimental, and clinical domains to establish NAMs as reliable tools for mechanistic IBD research and therapeutic screening.

## 6 Conclusion

The evolution of IBD research is shifting decisively towards more human-relevant, mechanistically informative, and ethically conscious models. Next-generation non-animal models, including advanced *in vitro* systems and computational tools, are redefining how we understand and investigate complex pathophysiological processes in IBD. These methodologies not only align with the principles of the 3Rs but also provide higher predictive power, especially when integrated with multi-omics data and patient-specific insights. However, their widespread adoption is contingent upon overcoming current limitations such as the lack of immune system and microbiota integration, technical complexity, and the need for regulatory validation. As interdisciplinary collaborations continue to grow, NAMs hold the potential to become indispensable tools in IBD research, therapeutic discovery, and translational medicine.

## Declaration

The authors declare that they have no conflicts of interest, financial or otherwise, that could have influenced the outcomes of this study.

## Consent for publication

All authors listed have approved the manuscript before submission, including the names and sequence of authorship.

## CRediT authorship contribution statement

**Priyanka Raju Chougule:** Writing – review & editing, Writing – original draft, Visualization, Software, Investigation, Conceptualization. **Sukesh Narayan Sinha:** Writing – review & editing, Supervision, Conceptualization.

## Declaration of competing interest

The authors declare that they have no known competing financial interests or personal relationships that could have appeared to influence the work reported in this paper.

## Data Availability

All the information in the manuscript is accessible. Upon a justified inquiry, the corresponding author shall furnish the datasets utilised or examined in the course of the present study.
